# Supplementary material for: Clinician Recommendation for Hereditary Genetic Testing in Participants at Increased Risk for Hereditary Cancer
Source: Cancers (Basel). 2025 Jun 14;17(12):1994. doi: 10.3390/cancers17121994 (PMC12190242; doi:10.3390/cancers17121994)
Supplement: Supplementary file 1 [file cancers-17-01994-s001.zip › cancers-3593172-supplementary.pdf]

Reported Cancer Types, could select >1

Cancer Types	n	%
Blood	129	4.5%
Breast	640	22.2%
Endometrial	129	4.5%
Gastrointestinal (GI)	336	11.7%
Head & Neck	58	2.0%
Ovarian	87	3.0%
Prostate	312	10.8%
Pancreatic	142	4.9%
Renal	82	2.8%
Prostate	312	10.8%
Skin	546	19.0%
Thyroid	70	2.4%
Other types reported	35	1.2%
Total	2878	100.0%
